# SNP Discovery with EST and NextGen Sequencing in Switchgrass (*Panicum virgatum* L.)

**DOI:** 10.1371/journal.pone.0044112

**Published:** 2012-09-25

**Authors:** Elhan S. Ersoz, Mark H. Wright, Jasmyn L. Pangilinan, Moira J. Sheehan, Christian Tobias, Michael D. Casler, Edward S. Buckler, Denise E. Costich

**Affiliations:** 1 Institute for Genomic Diversity, Cornell University, Ithaca, New York, United States of America; 2 Plant Breeding and Genetics, Cornell University, Ithaca, New York, United States of America; 3 Joint Genome Institute, Department of Energy, Walnut Creek, California, United States of America; 4 USDA-ARS, Western Regional Research Center, Albany, California, United States of America; 5 USDA-ARS, U.S. Dairy Forage Research Center, Madison, Wisconsin, United States of America; 6 USDA-ARS, Robert Holley Center, Ithaca, New York, United States of America; East Carolina University, United States of America

## Abstract

Although yield trials for switchgrass (*Panicum virgatum* L.), a potentially high value biofuel feedstock crop, are currently underway throughout North America, the genetic tools for crop improvement in this species are still in the early stages of development. Identification of high-density molecular markers, such as single nucleotide polymorphisms (SNPs), that are amenable to high-throughput genotyping approaches, is the first step in a quantitative genetics study of this model biofuel crop species. We generated and sequenced expressed sequence tag (EST) libraries from thirteen diverse switchgrass cultivars representing both upland and lowland ecotypes, as well as tetraploid and octoploid genomes. We followed this with reduced genomic library preparation and massively parallel sequencing of the same samples using the Illumina Genome Analyzer technology platform. EST libraries were used to generate unigene clusters and establish a gene-space reference sequence, thus providing a framework for assembly of the short sequence reads. SNPs were identified utilizing these scaffolds. We used a custom software program for alignment and SNP detection and identified over 149,000 SNPs across the 13 short-read sequencing libraries (SRSLs). Approximately 25,000 additional SNPs were identified from the entire EST collection available for the species. This sequencing effort generated data that are suitable for marker development and for estimation of population genetic parameters, such as nucleotide diversity and linkage disequilibrium. Based on these data, we assessed the feasibility of genome wide association mapping and genomic selection applications in switchgrass. Overall, the SNP markers discovered in this study will help facilitate quantitative genetics experiments and greatly enhance breeding efforts that target improvement of key biofuel traits and development of new switchgrass cultivars.

## Introduction

Switchgrass (*Panicum virgatum*L.) is a perennial C4 warm-season grass native to North America, where it occurs naturally from 55° N latitude to deep into Mexico, mostly as a dominant species of the tall grass prairies. In North America, it has been used for more than 50 years for soil conservation, as a forage crop, and as an ornamental grass [1]. In 1992, switchgrass was designated by the United States Department of Energy (DOE) as a model herbaceous energy crop for ethanol and electricity production, selected out of a wide array of candidate species [2]. Switchgrass possesses many desirable qualities of a biomass crop for energy and fiber production, including high-net biomass production per hectare, low production costs, low nutrient requirements, relatively low ash content, high water use efficiency, extended range of geographic adaptation, ease of establishment by seed, adaptation to marginal soils, and potential for carbon storage in soil [3]–[5].

Two genetically and phenotypically distinct switchgrass ecotypes, lowland and upland, were identified in early genetic screening studies. They are distinguished by a number of morphological traits and their natural habitat. The lowland ecotype has a taller, coarser, upright phenotype, with a more rapid growth habit compared to the upland ecotype, and is generally found in wetter habitats, such as floodplains. The upland ecotype is found in drier sites and is recognizeable by its finer stemmed, and often semi-decumbent phenotype [1], [6]. With respect to genetic distinguishing features such as ploidy levels, lowland switchgrass ecotypes are mostly tetraploid (2n = 4x = 36), whereas upland switchgrass ecotypes are much more complex in their ploidy levels, and generally display higher orders of ploidy. Upland ecotypes, despite the high frequency of octoploidy (2n = 8x = 72), also show a high incidence of aneuploidy and both tetraploid and hexaploid chromosome numbers, with rare reports of diploidy and even duodecaploid individual plants were reported [7]–[11]. Due to the differences in ploidy levels, these two ecotypes are mostly reproductively isolated with only occasional gene flow. However, the level of natural gene flow between the ecotypes is unknown. Although most of the recent research and breeding has focused on the lowland ecotype, with its stable, simpler genome and its high yield potential, particularly in warmer parts of the US, our project targeted northern-adapted, upland germplasm.

The molecular genetic characterization of switchgrass began with the use of both restriction fragment-length polymorphisms (RFLP) and randomly amplified polymorphic DNA (RAPD) markers to develop genetic fingerprints for the existing cultivars [9], [12]. These works established that the upland and lowland ecotypes were genetically distinct from one another, based on chloroplast and nuclear DNA markers. The natural distribution and history of switchgrass suggest that the species most likely possesses high levels of genetic variation. At higher ploidy levels that prevail in upland-adapted germplasm, polyploidy and polysomic inheritance patterns may contribute to this diversity. Observed frequencies of multivalents [13], [14] and increased levels of within-cultivar diversity observed in octoploids relative to tetraploids tends to favor this view [15]. Although prior studies highlighted a need for molecular maps to assist and hasten breeding efforts on primary biofuel traits, preliminary marker studies in the 2000 s determined that mapping would not be straightforward [16]. Starting in 2006, with the new wave of interest in genetic improvement for biofuel production through marker-assisted breeding and genomic selection, the DOE funded several new projects under the Biomass Genomics Research Program [17]. To date, three of these projects targeted several switchgrass cultivars for EST and short-read genomic sequencing [18] for the purpose of marker development to promote future efforts for quantitative genetic practices such as linkage mapping, association mapping, and genomic selection.

The massive natural distribution and outcrossing life history of switchgrass suggest that the species most likely possesses high levels of genetic variation. Domestication efforts targeting improvement of the feedstock characteristics of switchgrass have only been in progress for the last two decades. Therefore, in many regards, switchgrass is an undomesticated forage grass that has held a dominant ecological role in large parts of the US prairie. This suggests that even registered cultivars are likely to have retained considerable allelic variation that could be utilized to improve the biofuel production potential and efficiency of this species. Conventional breeding efforts of switchgrass are time consuming and challenging. Marker-assisted breeding could reduce the cycle time of this perennial by severalfold. However, assuming it has characteristics similar to other highly outcrossing grasses, such as maize, the traits targeted for domestication/breeding are very likely to be controlled by hundreds of quantitative trait loci (QTL) with small effects [19]. In a breeding context, these numerous small-effect QTL are best utilized using a marker-assisted breeding approach known as “Genomic Selection” (GS) or “Genome-wide selection”(GWS). GS relies on a simple principle of marker-trait association: When thousands of markers spanning the whole genome have been tested together for their association with a trait, at least one marker will be in linkage disequilibrium (LD) with each and every QTL regardless of the effect size or the location, whereby all QTL effects can be captured [20]. In low diversity species like cattle, this can be achieved by as few as 50,000 SNPs [21]. However, in a high diversity species like maize, more than two million SNPs may be necessary [22] to properly carry out GS across the species. Regardless of the species, however, all GS studies require preliminary studies with a large-scale, marker-discovery component. Such efforts are already underway forseveral other organisms such as *Arabidopsis thaliana* [23], rice [24], and maize [22], [25]. For switchgrass, assuming it would be similar to maize, it is likely that GS efforts will also require a large number of markers, estimated between 50,000 and two million SNPs. The actual number will be determined by the effective population size, effective recombination rate, trait heritability, and number of QTL, which may be highly variable between individual breeding programs.

Next generation sequencing platforms are transforming the way genomes are analyzed. Although SNP discovery using short-read sequence data is still in its early stages, several studies have already demonstrated that large numbers of high quality SNPs can be identified in a cost-effective manner using these data [26]. In these studies, deep-sequence coverage across many samples was necessary to identify high-quality SNPs. One way to achieve high levels of overlapping coverage between the libraries is to reduce the number of genomic sites surveyed in each library, which would allow for deep sequencing over selected fractions of the genome. This can be achieved by digesting each sample with a common restriction enzyme, often with a DNA-methylation state bias to enrich for transcribed regions and generate reduced representation genomic libraries (RRGLs) [27]–[29].

Here we describe how we coupled EST library and short-read sequencing approaches to discover over 149,000 SNPs in switchgrass. In the process, we developed a consensus-reference sequence of the switchgrass transcriptome of about 87.5 Mbs spanning the gene space of the switchgrass genome to anchor RRGL reads. Furthermore, we investigated population structure within our samples through PCA analysis on ∼4400 SNPs that had complete genotype information across all samples.

## Methods

### Plant material

Switchgrass cultivar seeds and clones were provided by MDC (Table 1, see Table S2 for the library names by cultivar). All plants were grown in a greenhouse at Cornell University, Ithaca, NY, in a soilless potting mixture, under ambient light conditions, watered as needed, and fertilized once weekly with 300 ppm 21–5–20 NPK solution. To mimic the natural vernilization conditions and enhance germination rates, the seeds were incubated in damp soil in a 40°F cold room for 2–6 weeks prior to planting in the greenhouse.

**Table 1:** Switchgrass cultivars, breeding populations and linkage population parents used as sources of RNA and DNA for EST and SRSL libraries.

Line Name	Population Type
Blackwell	Upland cultivar
Carthage	Upland cultivar
Cave-In-Rock	Upland cultivar
Dacotah	Upland cultivar
Forestburg	Upland cultivar
Kanlow	Lowland cultivar
KY1625	Upland cultivar
Pathfinder	Upland cultivar
Shelter	Mixed
Sunburst	Upland cultivar
WS4U	Upland “4X” germplasm pool
WS8U	Upland “8X” germplasm pool
WS98-IP	NA
WS98-SB	NA

Once the plants reached eight-leaf stage, leaf tissues were sampled for DNA and RNA library preparation. To allow for destructive sampling at an early developmental stage, each individual was propagated with multiple rooted cuttings prior to sampling. Each library was prepared from leaf-tissue samples from a single clone of a single seed parent, which was sampled from a greenhouse-grown population of several seed parents from the same seed lot.

### EST libraries and unigene clusters

Total RNA was isolated from 13 different cultivars of switchgrass as described in the plant material section, using the standard TRIzol® protocol (Invitrogen, Carlsbad, CA). A list of cultivars and libraries made is shown in Table S2.

### cDNA library construction and sequencing

Switchgrass poly-A RNA was isolated from the total RNA using the *Absolutely mRNA Purification Kit*™ from Agilent (Palo Alto, CA) according to themanufacturer's instructions. Poly-A RNA purity and quantity was assessed with an *Agilent Bioanalyzer™* (Agilent, Palo Alto, CA). First-strand cDNA was generated using a *Creator SMART cDNA Synthesis Kit*™ (Clontech, Mountain View, CA), according to the manufacturer's protocol. For each cultivar's first-strand cDNA synthesis, 1 µg of poly-A RNA, SMART IV Oligo anda CDS-3M adapter (TRIMMER-DIRECT cDNA Normalization Kit, Evrogen, Moscow, Russia) that incorporates asymmetric *Sfi*I restriction enzyme sites (*Sfi*IA and *Sfi*IB) at the 5′ and 3′ ends of cDNA were used. First-strand cDNA was amplified by a long-distancePCR (LD-PCR) protocol with 15 PCR cycles: 94°C for 7 seconds, 66°C for 30 seconds, 72°C for 6 minutes. Normalization was accomplished by using a Trimmer-Direct cDNA Normalization Kit™ (Evrogen, Moscow, Russia) according to the manufacturer's protocol. Briefly, 1µg of amplified cDNA was purified with Qiagen PCR Purification Kit™ (Qiagen, Valencia, CA), precipitated with ethanol, and dissolved in nuclease free water. cDNA was mixed with 4x hybridization buffer, overlaid with mineral oil, denatured at 98°C for 3 minutes and allowed to renature at 68°C for 5 hours.Double stranded nuclease (DSN) treatment was performed as described in the Evrogen kit manual. The ssDNA fraction remaining after DSN treatment was amplified with primers M1 and M2 for 18 cycles (94°C for 7 seconds, 66°C for 30 seconds, 72°C for 6 minutes), followed by digestion with *Sfi*I restriction enzyme. After digestion, the library was size fractionatedto >0.5 kb. To create a normalized cDNA library, the digested cDNA was unidirectionally ligated into *Sfi*I-digested pDNR-LIB vector for *in situ* amplification in bacteria. ElectroMax T1 DH10B cells (Invitrogen, Carlsbad, CA)were transformed with the ligation mixture by electroporation. Via colony counts, the titer of the original library was determined to be about 3×10^6^ cfu/ml. Twenty-four colonies per transformation event were randomly picked and primers pDNR-LIB_forward andpDNR-LIB_reverse (primers designed specifically for adaptor sequences of the Evrogen kit) were used to amplify and verify the inserts. Micro-titer plates in 384 well format were used for sequencing the vector inserts in picked colonies from both ends on ABI 3730 instruments (Applied Biosystems, Foster City, CA) at JGI labs in Walnut Creek, CA.

### EST sequence processing and assembly

ESTs from all libraries were processed through the JGI EST pipeline. ESTs were generated in pairs, using a 5′ and 3′ end-read from each cDNA clone. Common patterns at the ends of ESTs, such as vector and adaptor sequences, were identified and removed using a custom software tool developed internally at JGI. Clones were identified as “insertless” if more than 200 bases of vector sequence at the 5′ end or less than 100 bases of non-vector sequence remained in the sequence. Next, ESTs were trimmed for quality using a sliding window trimmer (window size  = 11 bases). Once the average quality score in the window was below the quality threshold (phred quality score of 15), the EST was split and the longest remaining sequencewas retained as the trimmed EST sequence, unless less than 100 bases of high-quality sequence remained, in which case, the sequences were removed from further processing. In the next step, ESTs that contained poly-A or poly-T tails were trimmed andretained unless the remaining sequence was shorter than 100 base pairs, in which case they were discarded. In the following step, ESTs consisting of more than 50% low-complexity sequence (even if it was good quality) were also removed from the final set of processed ESTs. In cases where more than one read from the same clone in which the same direction existed, the longest high-quality read was retained.

Sister ESTs (paired-end reads) were categorized as follows: if one EST was insertless or a contaminant, then, by default, the second sister was categorized as the same and was discarded. However, when retained, each sister EST was treated separately for complexity and quality scores. Lastly, an annotational quality check was conducted by comparing the EST sequences with those in the GenBank nucleotide database to identify contaminants, i.e., non-desirable sequences such as those matching non-cellular and rRNA sequences. Once identified, those sequences were removed from the final set of processed ESTs. For clustering, ESTs were evaluated with *malign*, a k-mer-based alignment tool [30], which clusters ESTs based on sequence overlap (k-mer  = 16, seed length requirement  = 32, alignment identity > = 98%). Clusters of ESTs were further merged based on sister ESTs using double linkage. Double linkage requires that two or more matching sister ESTs exist in both clusters in order to be merged. EST clusters were then assembled using CAP3 [31] to form consensus sequences.

Clusters may have more than one consensus sequence for various reasons, including alternative splicing, long-insert sequences, or errors in assembly. Cluster singlets are clusters of multiple reads from the same EST, whereas CAP3 singlets are single ESTs that had joined a cluster but, during cluster assembly, were isolated into a separate singlet consensus sequence. ESTs from each separate cDNA library were clustered and assembled separately and, subsequently, all of the ESTs for all cDNA libraries were clustered and assembled together. For cluster consensus sequence annotation, the consensus sequences were compared to Swissprot protein database using BLASTX and the annotations of the hits were reported.

### Illumina Genome Analyzer Sequencing

Genomes of one sample from each of 13 cultivars were sampled as described previously. The DNA libraries were digested with the methylation-sensitive restriction enzyme *Hpa*II and reduced-representation libraries (RRLs) were prepared as described in [29]. In this manuscript, these libraries are referred to asshort-read sequencing libraries or SRSLs. These libraries were sequenced as single-end 35 bp reads on a first-generation Illumina Genome Analyzer (Illumina-GA) by JGI labs. All sequences are submitted to GenBank, for sequence accessions see the supplementary information file Text S1.

### Assembly and SNP calling

Upon receipt of the sequences from JGI, each of the SRSLs was checked for reads that started with “CGG” sequence, the overhang left by the restriction enzyme upon digestion. Note that in cases where the second C of the CCGG recognition sequence is methylated, the enzyme cannot cut at the recognition sequence. Also, in cases where low-quality, broken molecules were included in the library, the read sequences will not contain the target overhang sequence. We also applied a prefilter to discard reads containing homopolymers larger than 16 bps. The resulting sequences were saved in fastq format and were analyzed with the *panati* suite of programs (http://panati.sourceforge.net/) [32].

Briefly, *panati* works as follows:

1. To enhance the search speed for mapping reads against the reference, the first step of the analysis process is building a sequence index from the reference sequence. This was implemented in the “*panati*-build” module, with user-defined options for “word-size,” i.e., the size of the words within the index, and “shift-size”, i.e., the length to shift the word window. Shift-size can be variable between 11 and 16 bps, where smaller shift-step size is slower but more accurate. We have used a 12 bps shift window.
2. The second step involves running the “fastq-qc” module on the SRSLs. This program takes in each read and begins to trim the sequence, starting at the 3′ end of the read, until a user-specified quality score threshold is reached. The minimum length required to include the trimmed read in the assembly can also be specified. Sequences are trimmed based on length and quality. For our specific case, we trimmed the 3′ end of the sequences until a minimum phred-like quality score of 10 or greater was established for the remaining sequence. After trimming, if the sequence size dropped below 12 bps, we discarded the read.
3. The next step was performing an assembly/alignment of the reads against a sequence index using a banded-Smith-Waterman algorithm [33]. We allowed two mismatches and up to three bp gaps. Note that these thresholds are based on average read length within the sequence library, which, in our case, was about 25 bps after trimming. We allowed multiple hits to the reference for the first round of assembly; however, if the multiple hits with these initial criteria could not be resolved in favor of a single alignment by evaluation of quality scores at the mismatch positions and the location of the mismatches within the alignment downstream, the read was discarded from further consideration for SNP calling.
4. The fourth step involved filtering the output of the previous step with user-defined criteria to improve the quality of the SNPs that would be reported from the final assembly, using a “combine-samples” program. Preliminary results indicated that the most important factorsaffecting the reliability of a SNP call were the number of reads that carry the alternate allele at the SNP position, as well as the total number of reads at that position. In an inbreeding species, detection of multiple alleles at the SNP position from a single library indicates to problems with assembly. However, switchgrass is highly heterozygous and is polyploid. Therefore, heterozygosity at aSNP position within libraries or, in other words, observation of a SNP within individual libraries is possible. To accommodate this characteristic of the species number of sequences required at the SNP position can be specified with two different run time flags: “-d”to specify the number of alternate read-carrying reads at the SNP position within a library, and “-l” to specify the number of alternate allele-carrying reads across the libraries. We called SNPs with various reliability thresholds for these options, but we are only reporting the results from the SNP callingwith a“–d 3–l 3” option, where we required at least three reads to carry the alternate allele at the SNP position. Further, *panati* allows forscreening of the SNP positions for the ratio of reference reads to alternate reads, and minimum read quality at the SNP position. For switchgrass, we set the ratio of reference to alternate allele to 1:7 to allow for the influences of dosage effects that can be created due to polyploidy, and set a minimum quality threshold of aphred-like quality score of 20.

We did not actively screen for transposon and retro-transposon sequences; however, by virtue of the way the alignments and SNP calling are performed with *panati,* it filters reads that map to multiple locations. This feature of *panati* was used as a passive screen against influences of repetitive sequences during assembly.

The resulting files were distributed from our website [32]. The *panati* parameters used for generating each result file were described with the files on the website.

### Genotype calling

We called genotypes from the raw-read data as follows. A genotype was called only if the read depth for an individual at the SNP locus was greater than or equal to four reads. For the purpose of genotype calling, we treated the SNP data as if they were dominant-marker data. Individuals were called homozygous if they carried four or more reads for one allele and no reads for another allele. Individuals with four or more reads carrying both alleles were called heterozygous. SNPs with missing genotypes were excluded.

### Population Structure Analysis by PCA

Principal-components analysis (PCA) was performed using R statistical software package on the genotypes obtained as described above. A set of SNPs that had complete genotype information across all lines was used (4400 SNPs) for the analysis. The SNPs identified were re-coded numerically as 0 for reference allele, 2 for alternate allele and the ratio of reference allele to alternate allele for the heterozygote value.

### SNP calling from ESTs

SNPs from the ESTs that were initially used to construct the unigene-cluster consensus sequences (approximately 110,000 sequences) were called by aligning the individual ESTs (about 600,000 sequences) against the reference sequence used for the SNP calling with SRSLs. We used *panati* for this procedure, with two reads at the SNP position requirements (d −2, l−2) for calling a SNP.

### Software availability

The manuscript on *panati* is currently under review. The program is available upon on request by contacting MHW directly or the corresponding authors. The other software tools used for the study are available by contacting the corresponding authors.

## Results

The most significant challenge for SNP discovery in a species without a fully sequenced genome using short-read sequencing is the lack of a reliable *de novo* assembly algorithm that can operate without a reference sequence to anchor the individual reads. Although several *de novo* assembly algorithms were released for public use in the last few years, [34]–[36] none have yet been shown to work for highly complex plant genomes with heavily repetitive sequences.

Although it is possible to anchor some portion of short-read sequences to a related taxon for which a complete genome sequence is available, this is undesirable for SNP discovery. This is due mostly to confounding of the polymorphisms that discriminate species from each other (i.e. between species divergence) and are not segregating within the target species. This also manifests as an inability to identify the reference alleles (except for the rare occasion where a shared polymorphism exists between two species). Computationally, SNP discovery algorithms often prevent SNP calling against the reference sequence when no reads representing the reference allele are present in the assembly. Therefore, our initial objective for this project was to generate a within-species reference genome sequence, a DNA consensus sequence that would provide a scaffold to map short-read sequences for SNP discovery.

There are many possible approaches for generating a genomic sequence scaffold (i.e. shotgun Sanger sequencing, 454 sequencing, and RRGL sequencing), but the fastest, least expensive, and most popular method is to establish a transcriptome scaffold using EST libraries. The previous EST sequencing efforts by Tobias et al. [37], [38] provided about 500,000 publicly available sequences from multiple upland and lowland switchgrass cultivars. These ESTs were combined with approximately 100,000 EST sequences generated in this project from 13 upland cultivars or breeding populations of switchgrass, including both tetraploid and octoploid individuals (**See** **Table** **1** **- Plant Material**). Since the contribution of any individual cultivar to the EST sequence pool other than Kanlow was low, these sequences were not optimal for identifying differences between cultivars. However, they did provide sufficient coverage across the genome to allow us to createthe required scaffold sequence and to verify the SNP discovery procedure.

**Figure 1:**
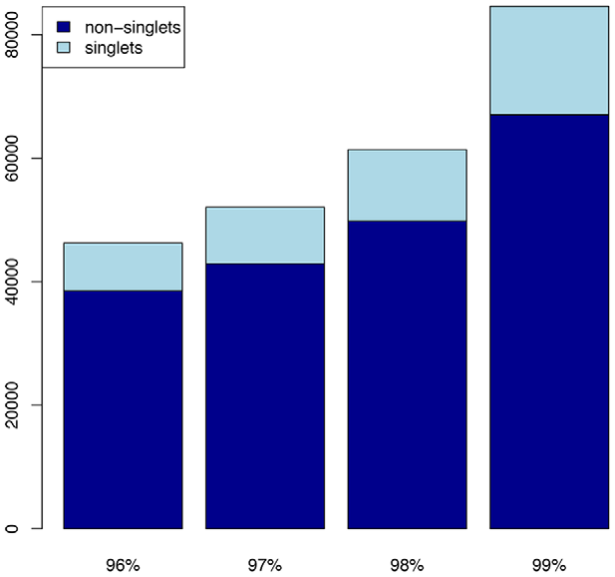
Determination of the % sequence identity threshold for EST assembly. In order to identify this threshold we have done EST clustering with a series of percent sequence identities, to determine the %identity that will cluster the sequences from individual loci together without splitting alleles at a loci. The jump observed in the cluster number between 98% and 99% sequence identity indicates that at locus level, sequences will be 98% identical, while the alleles at a locus are expected to be 99% identical.

Our next challenge was optimization of the sequence-clustering algorithm and clustering parameters to address issues created by high levels of paralogy in a polyploid genome. The severity of this challenge was conditional on the nature of the polyploidy, i.e. auto-versus allo-, which created whole or partial genome duplications. By adjusting the EST clustering parameters, it was possible to empirically test stringency levels required for the assembly. The optimum threshold was where clusters did not include paralogous sequences, but also did not split allelic variants coming from the same locus. We tested a series of thresholds of percent identity to determine how a variation in this parameter affects clustering. We expected to see a steady increase in the number of clusters up to the level where alleles would start to split, at which point a spike in the total cluster numbers and a reduction in the average number of reads per cluster would occur. In fact, the rate of increase in the number of consensus sequences generated showed a considerable jump between the 98% and 99% identity thresholds (**Table** **2** **,** **Figure** **1**). Therefore, for the SNP discovery experiments, we used the cluster consensus-sequence file created at 98% sequence identity for our reference sequence. The total length of the reference sequence was approximately 87 Mbps, including both EST assemblies from more than one read (cluster consensus) and EST sequences encountered only once (singleton sequences) (**See Table S1- coverage statistics**). The output from the EST clustering of all of the libraries is available at http://www.maizegenetics.net/snp-discovery-in-switchgrass [39].

**Table 2:** EST sequence clustering statistics at four levels of percent-identity thresholds.

	99%	98%	97%	96%
Number of consensus sequences	128,311	109,181	104,372	102,379
Number of Clusters	84,573	61,362	52,049	46,243
Number of Clusters (not singlets)	67,029	49,786	42,894	38,490
Number of Clusters (singlets)	17,544	11,576	9,155	7,753
Number of Cons sequences (not singlets)	84,547	72,804	69,414	67,593
Number of Cons sequences (singlets)	43,764	36,377	34,958	34,786
Number of Cons sequences/cluster	2	1.78	2.01	2.21
Number of Cons sequences/cluster (no singlets)	1.26	1.46	1.62	1.76
Longest number of cDNAs/cluster	23,938	14,150	32,729	50,844

**Figure 2:**
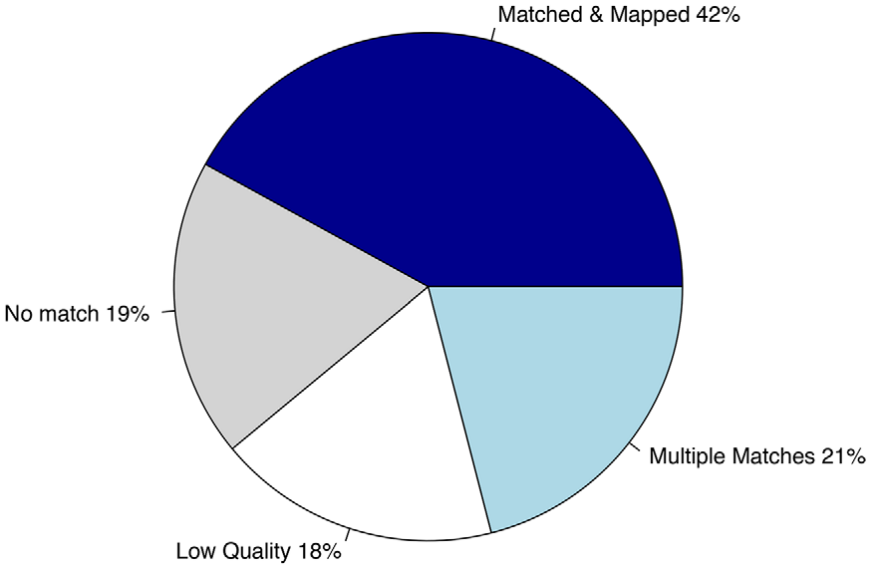
Distribution statistics for the assembly of reads obtained from SRSLs. 42% of the reads were matched and mapped while 19% of the reads found no match. Of the remaining 39%, 18% was low quality and 21% had multiple matches across the genome. This fraction was not used for SNP calling.

**Figure 3:**
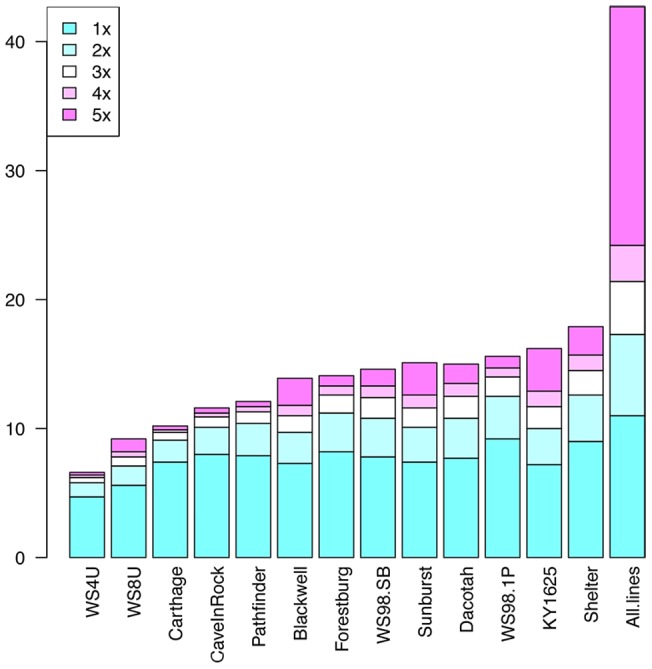
The depth of coverage over the reference sequences (87.5 Mbps) by cultivars, and across all cultivars. The fraction of the reference covered in each cultivar's individual sequence library was largest at 1x, and was decreasing in size as the coverage increased. However, when all the libraries were compiled together, although the size of the 1x fraction stayed more-or-less the same compared to individual libraries, the fraction that is covered at higher depths increased dramatically.

**Figure 4:**
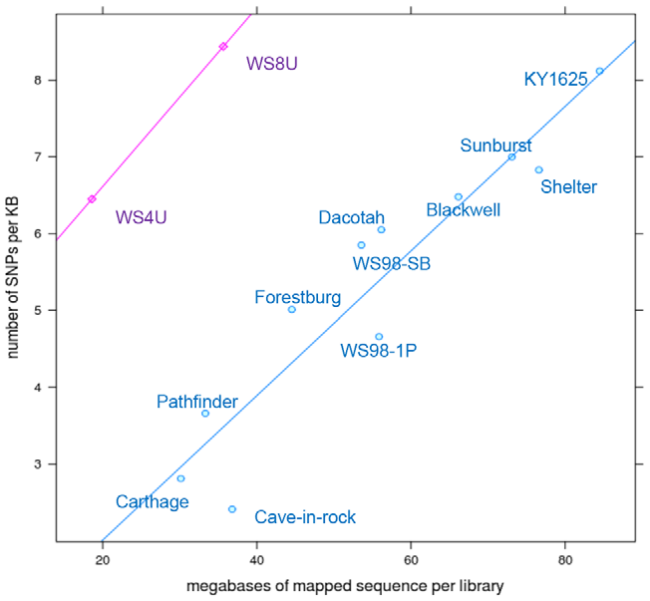
The correlation between levels of heterozygosity within individual libraries versus breadth wise coverage of the reference sequences. The correlation coefficient (R^2^) of the blue line is 0.89 while the correlation coefficient for pink line (R^2^) is >0.95.

SRSL coverage varied significantly across cultivars. Overall, 42% of the libraries were mapped to a single position against the reference, while about 39% could not be mapped but were found within the reference sequences. (On average, 18% of the sequences were low quality, and the remaining 21% had multiple equivalently strong matches on the reference and, therefore, were excluded from further analysis.) The remaining 19% were not found within the reference sequences available (**Table** **3** **-** **Figure** **2** **- mapping efficiency**). Since these sequences were identified as belonging to plant taxa [the possibility of cross-sample contamination during sequencing with another plant sample in the sequencing lab was disregarded], the 19% was deemed to be a portion of the switchgrass genome that wasn't covered with the EST libraries. For individual libraries, the largest reference coverage (at 1x sequence depth) was 17.9 Mbps (Shelter) and the smallest was 6.7 Mbp (WS4U) of total sequence. The SNP calling was restricted to the bases where at least three sequences carrying the alternate allele were observed (positions covered with at least 3x depth), and the ratio of reference to alternate alleles was no less than 1:7 and no more than 7∶1 (assuming octoploidy, i.e. a maximum of eight copies of a given allele at any given locus). This crudely compensated for the possible dosage effects of mainly tetra- or octo- ploid genomes, and prevented SNP calling due to sequencing or assembly errors. Across the libraries, total reference-sequence coverage was 42.7 Mbps at 1x minimum depth and 25.4 Mbpsat 3x minimum depth (**Figure** **3** **- library sizes**), the depth level required for SNP calling. As expected, more SNPs per Kb were detected from the libraries with higher coverage (average  = 5.73 SNPs/Kb), and thus the contribution of each library to the total SNP pool varied based on its size. Two exceptions to this trend were WS8U (8.37 SNPs/Kb) and WS4U (5.96 SNPs/Kb), which displayed more SNPs per Kb than any of the other cultivars compared to their library sizes (**Figure** **4** **- SNPs/Kb by library size**) (See Supplementary Table S1- coverage statistics). Since the amount of heterozygosity in a polyploid genome is expected to be high, we performed SNP calling in two stages, first from each individual library, which we followed with SNP calling from combined reads across all the libraries for consensus SNP calling (see Materials and Methods). Using this methodology, we identified a total of 149,502 SNPs and short (1–3 bps) indels.

**Table 3:** Total number of reads generated from each library and the mapping efficiency.

Line	Estimated Genome Size in MBps§	Total	% Final-mapped	% Unmapped	% Discarded
Blackwell	588.000	4,142,351	45.63%	34.40%	19.97%
Carthage	599.025	2,254,301	38.20%	44.62%	17.18%
Cave-in-rock	645.575	2,677,852	39.26%	43.13%	17.61%
Dacotah	581.875	3,527,502	45.48%	35.04%	19.48%
Forestburg	580.650	2,891,721	44.01%	37.17%	18.82%
KY1625	601.475	5,372,097	44.94%	35.53%	19.53%
Pathfinder	584.325	2,419,059	39.35%	43.67%	16.98%
Shelter	614.950	5,230,735	41.84%	40.23%	17.93%
Sunburst	612.500	4,742,716	44.03%	36.85%	19.12%
WS4U	591.675	1,330,787	39.92%	43.29%	16.80%
WS8U	556.150	2,324,402	43.82%	36.58%	19.60%
WS98-IP	602.700	4,021,678	39.67%	43.21%	17.11%
WS98-SB	546.350	3,439,133	44.50%	36.34%	19.16%

§ Values are calculated using the pg estimates reported in Costich et al. 2010 Table 2 with the conversion factor of 980 Mbps per pg per 2c nucleus value, divided by the ploidy level.

### Population structure and validation of the discovered SNPs

**Figure 5:**
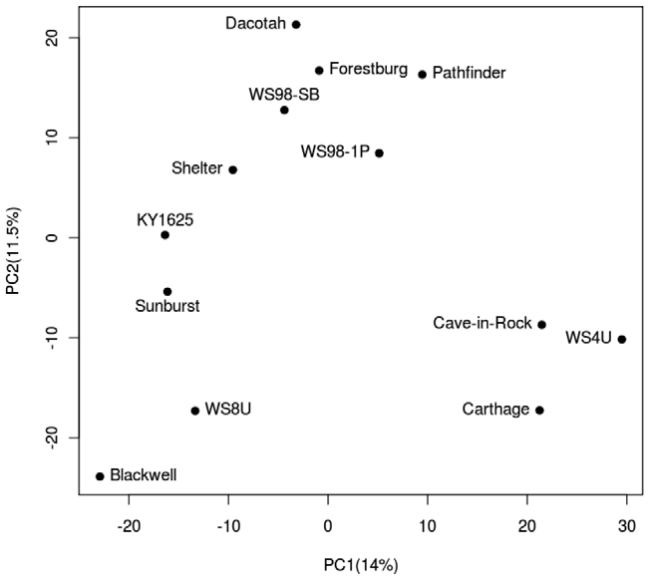
Principal Components Analysis (PCA) on 4400 SNPs where complete genotypes across all 13 samples were available. First two components explains about 25.5% of the variance. There is no apparent clustering tendency except for the lines at the bottom right corner: Cave-in-rock, Carthage and WS4U.

A small set of SNPswere identified in all 13 lines (4400 SNPs). These were used in a principal components analysis (PCA) to examine population structure in our sample (**See** **Figure** **5** **- PCA for lines**). No immediate patterns were detected from plotting the first two principle components, which together explained about 25% of the observed variance. PC1 explained about 14% of the observed variance, while PC2 explained the additional 11%.

As a preliminary validation for the SNPs called from short-read libraries, we attempted to compare SNPs discovered from EST sequences to the set of SNPs called from short-read sequences. Due to low depth of the EST libraries, and unbalanced library sizes for different cultivars, SNP discovery from the EST set was challenging. In addition, due to unequal reference-sequence coverage between the two library types (ESTs vs. SRSLs), the overlap between SNPs was expected to be small since transcriptome coverage from a single tissue type is expected to be a small fraction of the genome. As expected, the EST sequences covered over 99% of the reference sequence (transcriptome assembly) at 1x coverage (**Table** **3**), while, at 1x minimum depth, SRSLs covered about 48% of the available transcriptome reference, and only about 29% of the coverage that was 3x and over was considered for SNP discovery (see SNP declaration criteria in the Methods section), summing up to only about 29% overlap between the comparable fraction of SNPs detected by the two methodologies. With the requirement of a minimum of two reads with the alternate allele at each position (coverage at 2x depth), 24,436 SNPs were detected from the EST alignments (6.3 SNPs/Kb). However, since the read depth at each SNP position is lower in the EST alignments, these SNPs were considered lower quality than the SNPs discovered from the SRSLs. We presented these SNP data sets with the other data sets on [39].

## Discussion

Until recently, large-scale marker discovery studies have usually concentrated on a small number of organisms with sequenced genomes. With decreasing costs for DNA sequencing and genotyping, coupled with improved NextGen sequencing technologies, we anticipate growing interest in moving rapidly toward large-scale marker discovery and, eventually, genomic selection (GS) studies in organisms for which relatively little genetic data currently exist. There are many orphaned crop species in need of genetic resources, and thousands of germplasm accessions in repositories worldwide that need their genomes indexed to accelerate breeding programs. In the present study, we provide a framework for rapidly and cost-effectively moving from few genetic resources to genome-wide characterization of a species. This would be particularly useful for species that are of cultural or niche-market interest, and that are unlikely to receive extensive funding.

We generated 1.6 Gigabases (Gb) of DNA sequence data (Table 3), and mapped about 0.6 Gb (42%) back to the reference sequence of approximately 87 Mb. The effective size of the switchgrass genome was estimated to be about 550 Mbp (our calculations according to [7]), and if our libraries were completely random, we expected an average of 3x coverage over the genome with the number of reads generated. Since the reference sequence was estimated to be less than 16% of the overall genome sequence, despite an expectation for enrichment toward genic sequence representation within the SRSLs; it was somewhat surprising that we were able to map 42% of the reads acquired from SRSLs back to this presumably ∼15% (assuming 550 Mb genome) region of the genome. For an outcrossing grass species, it is common for the genome to be composed primarily of transposable elements. For instance, only about 15–20% of the maize genome is genic sequences, and the remainder consists of transposable elements. If the genic portion of the switchgrass genome comprises about 30% (approximately 165 Mbp expected), similar to maize [40], this may offer an explanation for the high frequency of reads hitting the available reference sequence of 87.5 Mbp (∼15%).

To determine the level of enrichment for genic regions, we calculated the read fractions in each library that contained the *Hpa*II cut-site overhang. Although, on average, only about 25% of all reads contained intact CCG tags, overall, 42% of total reads library found a unique match against the reference sequence, and another 21% was mapped to multiple positions against the reference. This disparity may be due to loss of part or all of the CCG tags during library construction and sequencing. Another much simpler explanation may be that the mapping stringency is low- since we have allowed for 2 mismatches and upto 3 bp indels for mapping, summing up to a total of 20% mismatch allowance during mapping. This mapping stringency is lower than the stringencies used for most species for similar experiments.

Overall, we were unable to use 58% of the reads (19% not represented in the reference, 39% unmappable) for the assembly. This indicates 41% of the total reads, or approximately 18 million25 bp reads, totaling about 451 Mbp were matched back to 29% of the reference at 1x or higher coverage.

To make SNP calls, we used an in-house assembly and SNP calling algorithm named *panati* for several reasons: 1) it could incorporate unigene cluster reference sequences as opposed to requiring genome sequence data; 2) it could integrate both short-read sequence data as well as longer-read Sanger sequence data for SNP calling; 3) due to the high incidence of heterozygosity in the cultivars sequenced, it was necessary to have an algorithm that can call SNPs on reads from individual libraries; 4) due to the polyploid nature of switchgrass, a SNP calling algorithm with adjustable parameters for the expected frequency of the reference allele versus the alternate allele at SNP sites was required; 5) a scalable algorithm that could work for variable percent-identity levels across libraries was needed; and, 6) some of the published SNP-calling algorithms are not successful at distinguishing real SNPs from sequencing errors and, thus, resort to modifying the SNP-calling criteria based on quality scores and genotypic contingency tests (Myles et al., 2009, Gore et al. 2009) [24], [17]. Such criteria can readily be implemented in the SNP-calling algorithms to reduce the false-positive rate.

As a measure of diversity, we calculated within and across library SNP detection rates. We observed a positive correlation between the total number of mapped reads per library (reference coverage) and the number of SNPs detected per kilobase pairs (Kbps) (**Figure** **4** **, R^2^ = 0.89**). Notable exceptions were WS8U and WS4U, two accessions that showed significant enrichment for diversity despite relatively small library sizes. WS8U diversity was similar to that of KY1625, despite the KY1625 library having 2.5x more reads, and WS4U diversity was similar to that of Blackwell, even though its library has about 4x more reads. This is likely due to the fact that WS4U and WS8U are diverse germplasm pools, each originating from a wide array of prairie-remnant populations with common DNA content (ploidy), i.e., WS4U plants are “tetraploids” with DNA content approximately 3.0 pg/nucleus; WS8U plants are “octoploids” with DNA content approximately 6.0 pg/nucleus [41]. This indicates that these "germplasm pools" were successful at increasing heterozygosity within individuals that came from these pools. All 11 of the other cultivars in this panel are traceable back to a single source-identified, prairie-remnant population or a very narrow geographic region, such as a single county.

Our results did not show a plateau for the correlation between the library size and the diversity levels. This indicates that our largest diversity estimate of ∼8 SNPs/Kb still underestimates the amount of diversity contained within these switchgrass accessions. Deeper sequencing per library is required to more accurately assessthe levels of diversity/heterozygosity in switchgrass. With the available data, we could only estimate the amount of heterozygosity for each library using the correlation detected between the library size and the heterozygosity. Excluding the WS4U and WS8Ulibraries, with a minimum of 2x coverage at any SNP position required for SNP calling, if all libraries were sequenced as deeply as our largest library (KY1625 (2.4 million reads)), Cave-in-Rock would still have the lowest heterozygosity, with ∼5.5 SNPs per Kb, while Forestburg would be the most heterozygote, with 9.5 SNPs per Kb. That is about twice as high as what was observed in high diversity maize varieties.

We used a subset of SNPs to examine switchgrass population structure with principal-components analysis. The first two principal components together explained 25% of the variance. We did not detect any clustering pattern that would indicate population structure within switchgrass. Since there was prior data suggesting that there are at least three distinct geographic clades – Central Great Plains, Northern Great Plains, and Eastern Savanna – within upland switchgrass germplasm, the lack of clustering observed in our data may be attributed to the relatively small sample size of these 13 genotypes, combined with the higher within-population variance in comparison to the between-population variance observed previously in switchgrass [42].

## Supporting Information

Table S1 **Depth and breadth wise coverage statistics by cultivar and across cultivars between 0x to 5x coverage.** 0x designates the fraction of genome that is not covered. SNPs were called from the fraction that is either 3x in an individual library or 3x across libraries.
(XLSX)

Table S2 **Names of the libraries that were used to tag reads from each library in the data files distributed from the project website at** http://www.maizegenetics.net/snp-discovery-in-switchgrass **.**
(XLSX)

Text S1 **GenBank SRA and dbEST accessions for the sequences generated for the project.**
(DOCX)
