# Supplementary material for: SNP Discovery with EST and NextGen Sequencing in Switchgrass (Panicum virgatum L.)
Source: PLoS One. 2012 Sep 25;7(9):e44112. doi: 10.1371/journal.pone.0044112 (PMC3458043; doi:10.1371/journal.pone.0044112)
Supplement: Text S1 — GenBank SRA and dbEST accessions for the sequences generated for the project. (DOCX) [file pone.0044112.s003.docx]

SRA accessions for the Illumina data:

SRA023697 (Carthage)
SRA023696 (WS98-1P)
SRA023695 (Pathfinder)
SRA023694 (Forestburg)
SRA023693 (Dacotah)
SRA023692 (WS98-SB)
SRA023691 (Cave-in-Rock)
SRA023690 (Sunburst)
SRA023689 (Blackwell)
SRA023688 (KY1625)
SRA023687 (Shelter)
SRA023686 (WS8U)
SRA023684 (WS4U)

 EST files have been loaded into dbEST

file    #subs    accession range
----    -----    -----------------
CCTW    8610    HO244025-HO252634
CCTX    8950    HO252635-HO261584
CCTY    8792    HO261585-HO270376
CCTZ    8999    HO270377-HO279375
CFBY    10216   HO279376-HO289591
CFBZ    2567    HO289592-HO292158
CFCA    8397    HO292159-HO300555
CFCB    9145    HO300556-HO309700
CFHA    9347    HO309701-HO319047
CFHB    9912    HO319048-HO328959
CFHC    9774    HO328960-HO338733
CFHF    9267    HO338734-HO348000
